# Clinical Breast MR Using MRS or DWI: Who Is the Winner?

**DOI:** 10.3389/fonc.2016.00217

**Published:** 2016-10-28

**Authors:** Francesco Sardanelli, Luca Alessandro Carbonaro, Stefania Montemezzi, Carlo Cavedon, Rubina Manuela Trimboli

**Affiliations:** ^1^Utà di Radiologia, IRCCS Policlinico San Donato, San Donato Milanese, Milan, Italy; ^2^Dipartimento di Scienze Biomediche per la Salute, Università degli Studi di Milano, Milan, Italy; ^3^Dipartimento di Radiologia, Azienda Ospedaliera Universitaria Integrata, Verona, Italy; ^4^Dipartimento di Fisica Sanitaria, Azienda Ospedaliera Universitaria Integrata, Verona, Italy

**Keywords:** breast cancer, diffusion-weighted imaging, magnetic resonance imaging, proton MR spectroscopy, systematic reviews and meta-analyses

## Abstract

Magnetic resonance imaging (MRI) of the breast gained a role in clinical practice thanks to the optimal sensitivity of contrast-enhanced (CE) protocols. This approach, first proposed 30 years ago and further developed as bilateral highly spatially resolved dynamic study, is currently considered superior for cancer detection to any other technique. However, other directions than CE imaging have been explored. Apart from morphologic features on unenhanced T2-weighted images, two different *non-contrast* molecular approaches were mainly run *in vivo*: proton MR spectroscopy (1H-MRS) and diffusion-weighted imaging (DWI). Both approaches have shown aspects of breast cancer (BC) hidden to CE-MRI: 1H-MRS allowed for evaluating the total choline peak (tCho) as a biomarker of malignancy; DWI showed that restricted diffusivity is correlated with high cellularity and tumor aggressiveness. Secondary evidence on the two approaches is now available from systematic reviews and meta-analyses, mainly considered in this article: pooled sensitivity ranged 71–74% for 1H-MRS and 84–91% for DWI; specificity 78–88% and 75–84%, respectively. Interesting research perspectives are opened for both techniques, including multivoxel MRS and statistical strategies for classification of MR spectra as well as diffusion tensor imaging and intravoxel incoherent motion for DWI. However, when looking at a clinical perspective, while MRS remained a research tool with important limitations, such as relatively long acquisition times, frequent low quality spectra, difficult standardization, and quantification of tCho tissue concentration, DWI has been integrated in the standard clinical protocols of breast MRI and several studies showed its potential value as a stand-alone approach for BC detection.

## Introduction

Valuable results of breast magnetic resonance imaging (MRI) were first obtained 30 years ago, when sequences acquired after intravenous injection of a Gd-chelate as a contrast material were added to a non-contrast protocol (1). This approach was further developed as bilateral highly spatially resolved contrast-enhanced (CE) dynamic study, which is now currently considered superior for breast cancer (BC) detection to any other imaging technique with a reported pooled sensitivity and specificity of 93.2 and 71.1%, respectively (2), as also shown by studies on MRI screening of women at increased risk of BC (3–6). In fact, CE-MRI provides a physiopathological information that correlates with increased vascularity, vascular permeability, and interstitial space in malignant tissue, not available from mammography and ultrasound. This diagnostic modality has been shown to provide even better sensitivity for BC detection than new techniques such as digital breast tomosynthesis (7–9). As a consequence, CE-MRI has been recommended for a spectrum of indications by medical bodies such as the American College of Radiology (10), the European Society of Breast Imaging (11, 12), and the European Society of Breast Cancer Specialists (EUSOMA) (13). The debate is still open on preoperative MRI (14, 15), but new indications, such as nipple discharge (16, 17) and evaluation of lesions with uncertain malignant potential (so-called B3 lesions), are emerging (18).

However, MR directions other than dynamic CE imaging have been explored searching for a better specificity and a deeper insight into BC biology and metabolism, opening a wider and wider window for the so-called *non-contrast breast MR*. Of note, the clinical relevance of a *non-contrast* approach for breast MR has been recently fueled by the evidence of Gd accumulation in the brain after multiple administrations of contrast material (19, 20), which implies a word of caution for the use of Gd-based contrast at least in the case of screening of healthy subjects when the risk profile is not high.

Traditionally, unenhanced sequences were associated to CE sequences in a breast MRI protocol for the sake of appraising morphologic features for lesion characterization. Several signs, typically appreciated on T2-weighted images, can be associated with malignancy, the most important being peritumoral and prepectoral edema (21), hook sign, i.e., a hook-like spiculated dendrite from the lesion to the pectoral muscle (22), and necrosis sign, i.e., a hyperintense center in a hypointense lesion (23). Non-enhancing septations are instead usually associated with benign lesions such as fibroadenomas (24).

Two other non-contrast MR methods were mainly implemented for an *in vivo* study of breast tissues: proton MR spectroscopy (1H-MRS) and diffusion-weighted imaging (DWI).

Proton MR spectroscopy is a non-invasive technique assessing biochemical tissue properties. The presence of a resonance at 3.14–3.34 ppm, attributed to choline metabolites, such as free choline, phosphocholine, and glycerophosphocholine, is usually reported as total choline (tCho). An increased tCho has been detected in malignant breast lesions (25–31), as a result of complex metabolic alteration of biosynthetic and/or catabolic phosphatidylcholine-cycle pathways: the *de novo* biosynthesis of phosphatidylcholine *via* the Kennedy pathway and three different major catabolic pathways, contributing to phosphocholine and/or tCho accumulation (31).

The other non-contrast approach – DWI – is a way for characterizing tissue properties on the basis of the difference in the movement freedom of water molecules (diffusion) along multiple spatial directions, quantified *via* the measurement of the mean diffusivity and the apparent diffusion coefficient (ADC) (32). Breast malignant tissues show restricted diffusion and significantly lower ADC values compared with those of normal and benign breast tissues (33).

Many studies were published regarding clinical breast applications of MRS and DWI showing a potential for both techniques. The aim of this review is to look at secondary evidence, published between 2010 and 2015, on 1H-MRS and DWI of the breast as well as to try to define which of the two techniques is clinically emerging as a routine tool added to CE-MRI or also potentially usable as a stand-alone approach for cancer detection.

## Diagnostic Performance of 1H-MRS

The diagnostic performance of 1H-MRS of the breast, performed using the single-voxel technique, was evaluated by four systematic reviews (34–37) (Table 1).

**Table 1:** **Diagnostic performance of proton MR spectroscopy and DWI of the breast as evaluated in systematic reviews and meta-analyses published between 2010 and 2015**.

Included studies	Lesions/patients	Pooled sensitivity		Pooled specificity		Notes	Reference
		Point estimate (%)	95% CI	Point estimate (%)	95% CI		
**Proton MR spectroscopy (1H-MRS)**							
19	1198/1183	73	64–82	88	85–91	tCho evaluation: visual, SNR, 2 or 4 cutoff values	(34)
18	1169/NR	71	68–74	85	81–88	tCho evaluation: visual, SNR, variable cutoff values	(35)
16	1049/NR	74	70–77	78	73–82	tCho evaluation: visual, SNR, variable cutoff values	(36)
10	792/NR	74	69–77	76	71–81	tCho evaluation: SNR, 2 cut off	(37)
**Diffusion-weighted imaging (DWI)**
13	964/815	84	82–87	79	75–82	Heterogeneity among individual studies; subgroup analysis	(50)
14	1276/1140	86	80–91	76	67–83	Heterogeneity among individual studies; subgroup analysis	(2)
26	2151/2111	91	84–95	75	61–85	Including 11 studies using *b* values ≤600 mm^2^/s	(51)
26	2151/2111	89	85–92	84	78–89	Including 30 studies using *b* values >600 mm^2^/s	(51)

The pooled sensitivity ranged between 71 and 74%, and the pooled specificity between 78 and 88%. Baltzer and Dietzel also performed a subgroup analysis for mass and non-mass lesions on six studies obtaining a pooled sensitivity of 68 and 62%, and a pooled specificity of 88 and 69%, respectively (34), showing that especially for non-mass lesions, 1H-MRS has a too low diagnostic performance to be used in clinical practice. Another subgroup analysis was performed on seven studies by Wang et al. (37), showing that the area under the curve (AUC) at receiver operating characteristic (ROC) analysis was higher (92 vs. 89%) when tCho signal-to-noise ratio ≥2 was selected as a cutoff for malignancy.

All studies included in this meta-analysis (37) showed a high heterogeneity for both sensitivity and specificity, probably due to the large variety of MRS techniques and different field strengths used. Even though improvements from newer technology (such as 3-T magnets and multiple radiofrequency sources) were expected, the authors (37) concluded that none of these innovations significantly influenced the diagnostic performance, as year of publication showed no effect on the diagnostic performance.

Notably, small masses (between 5 and 10 mm in diameter) or foci (<5 mm in diameter) are commonly encountered on CE-MRI, depending on the clinical setting, a proportion of them turning out to be false positives. As a consequence, to avoid work-up (targeted ultrasound, needle biopsy) would be a great achievement. However, in this regard, 1H-MRS is scarcely useful. The large majority of studies did not include lesions smaller than 10 mm, thus limiting the applicability of 1H-MRS for characterization of small lesions and early BC diagnosis.

One relevant technical problem for 1H-MRS, especially important for small lesions, is the need for patient’s immobility during the acquisition, i.e., for 5–10 min or more (depending also on the time for the preliminary optimization checks), which also impacts on patient’s throughput and cost-effectiveness. Of note, a trend for a higher sensitivity of 1H-MRS was observed when acquisition is performed before contrast injection, due to a detrimental effect of ionic-charged contrast materials in both experimental and clinical settings (38). As lesion localization (the placement of the spectroscopic volume of interest) has to be made on CE images (especially for otherwise not visible small lesions), the majority of 1H-MRS studies were performed after contrast injection, potentially implying a suboptimal sensitivity. On the other hand, a major clinical application of 1H-MRS should be lesion characterization, typically arising immediately after the detection, implying that 1H-MRS should be performed immediately after CE-MRI, which usually lasts about 15–20 min. Thus, a time prolongation of additional 10 min increases the probability of patient’s movements, reducing the possibility of getting good quality spectra.

Moreover, again especially important for small lesions, to adapt the volume of interest to the lesion morphology is often not easy: cubic or anyway squared volumes of interest tend to include tissues (fat or gland) surrounding the lesion, determining volume contamination and resulting in reduced sensitivity (28). Lesion located posteriorly, close to the pectoral muscle or the thoracic wall, or superficially, near the skin, may provide spectra with low signal-to-noise ratio. Thus, even using 3-T magnets and multiple radiofrequency sources, in clinical practice about 20% of lesions finally result to be not evaluable with MRS (39).

**Figure 1:**
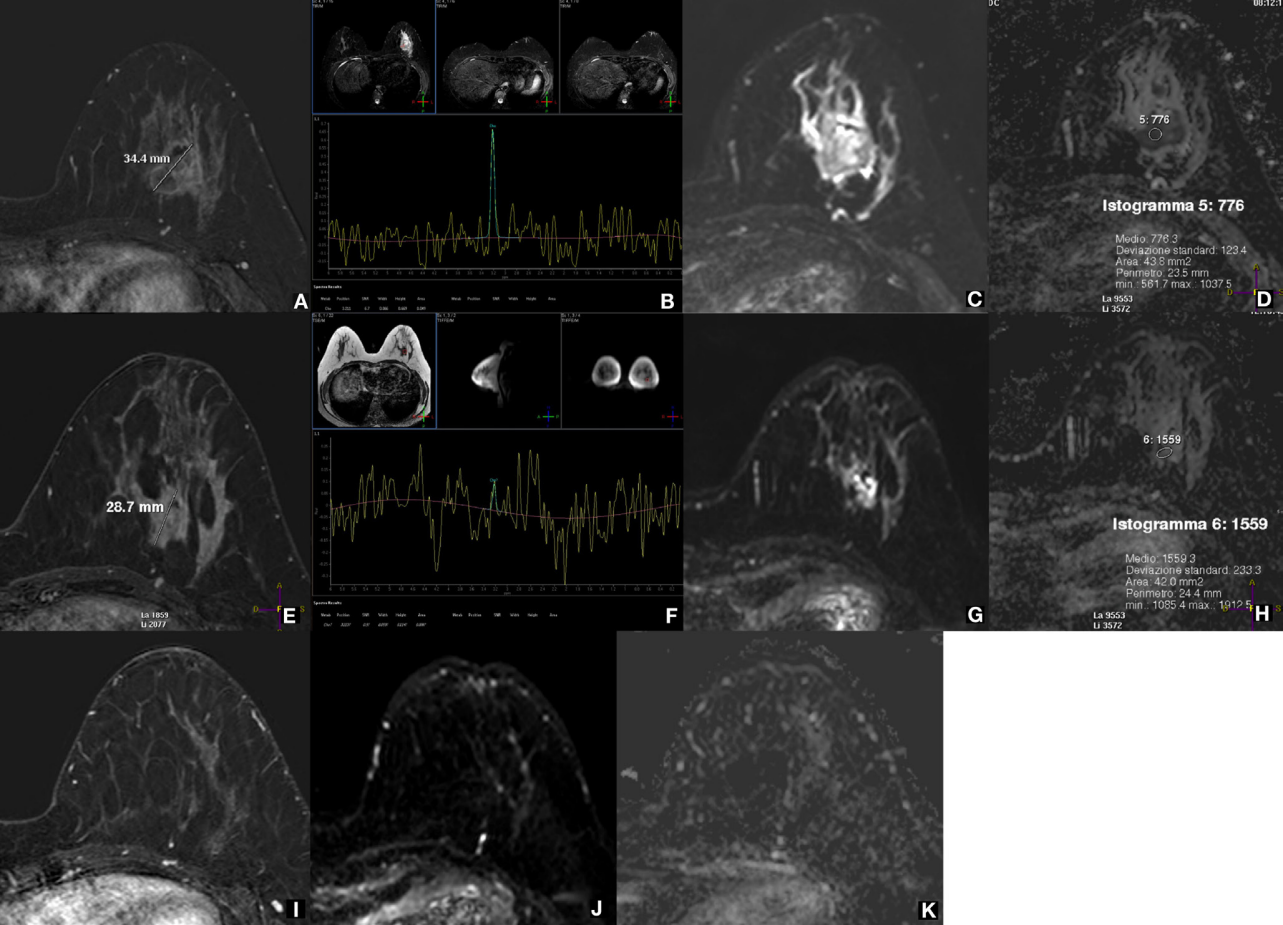
**1H-MRS and DWI for evaluating the response to neoadjuvant therapy (NAT) of a locally advanced breast cancer**. A 37-year-old woman with locally advanced breast cancer before, during, and after neoadjuvant therapy (NAT). Before treatment, the lesion at the lower external quadrant of the left breast is well depicted as a 34.4-mm nodule on subtracted CE-MRI with 0.1 mmol/kg of gadobenate dimeglumine **(A)**, shows a high tCho peak at 1H-MRS with a signal-to-noise ratio (SNR) of 6.7 **(B)**, low diffusivity as a high signal on DWI **(C)**, and a low (0.776 × 10^−3^ mm^2^/s) mean ADC value **(D)**. After two NAT cycles, the lesion is reduced in size (28.7 mm) at CE-MRI **(E)**, while the tCho peak is no longer detectable at 1H-MRS **(F)**; DWI **(G)** and ADC map **(H)** show an evident increased diffusivity (mean ADC 1.559 × 10^−3^ mm^2^/s). After the end of treatment, the lesion is not visible at all on CE-MRI **(I)**, DWI **(J)**, and ADC map **(K)**; therefore, MRS was not performed. The lesion was a metaplastic carcinoma with condroid differentiation (negative for estrogen, progesterone, and HER2 receptors, Ki67 80%), and after surgical removal, a complete pathological response was appreciated. Both 1H-MRS and DWI early predicted the pathological response to NAT showing an effect more pronounced than that of CE-MRI. Acquisition times: CE-MRI 9 min; 1H-MRS 8 min (including preparation); DWI 5 min (Philips Achieva STx 3.0 T, MultiTransmit radiofrequency technology; dedicated 16-channel breast coil; Azienda Ospedaliera Universitaria Integrata, Verona, Italy).

Several of these limitations are not acting when 1H-MRS is applied for the evaluation of the response to neoadjuvant therapy (NAT), typically administered to patients with locally advanced BCs. Studies have shown that 1H-MRS enables an early prediction of the final NAT effect (40–42). This is, in our opinion, the most promising clinical application of 1H-MRS, even though it regards a relatively small fraction of patients. However, also in this field, as we will explain below, DWI is a strong competitor (Figure 1). Anyway, for a well-accepted application of MRS techniques to the NAT setting, large multicenter studies using clearly standardized pathologic response classification are warranted (43–46).

Finally, we should note that 1H-MRS was mostly applied in clinical breast studies using methods not allowing for quantification of tissue tCho concentration. Both the SNR between tCho peak and noise (usually with a ≥2 threshold for malignancy) (34–37) and the integral under the tCho peak (30) have limitations and low potential for standardization. On the other hand, tissue tCho quantification using internal or external standard of reference (47) implies dedicated high technical expertise. This highlights the need for cooperation with a physicist experienced in MRS, not only for tCho tissue quantification but also generally if MRS has to be integrated in a clinical setting. This is a possibility that only large hospitals or academic facilities can afford and is another limitation for MRS effectiveness.

Highly interesting results were obtained, even at 1.5 T, by Stanwell et al. (48), carefully referencing the spectra using a special post-processing: 80% sensitivity and 100% specificity. They optimized spectral resolution from human breast tissues resolving the composite choline resonances of the tCho peak. False positives including three lactating women were distinguished by a resonance at 3.28 in contrast with the profile from BC patients consistent with phosphocholine centered at 3.22 ppm. However, a false negative rate of 20% remained unresolved, and this high spectral resolution was never reproduced, not even using 3-T magnets.

Looking at the potential prognostic value of 1H-MRS of the breast, it is worth to note that tCho levels, evaluated by 1H-MRS, were significantly higher for invasive ductal carcinoma, for cancers with high nuclear or histologic grade, and ER-negative and triple-negative status (49). This perspective deserves confirmation in future large studies with quantification of tissue tCho concentration.

## Diagnostic Performance of DWI

Breast MR sequences for DWI have been mainly used to try to characterize breast lesions, adding a new parameter in the algorithm for diagnosis, with the potential for avoiding unnecessary biopsy, especially for MRI-only visible lesions that should be sampled under MR-guidance, a time-demanding and high-cost procedure (11).

Studies were performed in order to understand the potential value of DWI added to CE-MRI. Two meta-analyses, from Chen et al. (50) and from Zhang et al. (2), reported an overall sensitivity of 84 and 86% and an overall specificity of 79 and 76%, respectively. However, a notable heterogeneity among individual studies was observed, and a subgroup analysis to investigate the source of this heterogeneity was performed. The two most important factors resulted to be the *b* value of diffusion gradient used and the so-called *threshold effect* (a detectable difference in sensitivities and specificities due to different thresholds used in included studies).

Different *b* values significantly affect the ADC of breast lesions and therefore confound quantitative DWI. In a large systematic review performed on 26 studies, including a total of 2111 patients with 2151 breast lesions, Dorrius et al. (51) showed that the combination of *b* = 0 and 1000 s/mm^2^ is optimal for benign/malignant differentiation. Pooled 89% sensitivity and 84% specificity were reported for *b* values >600 s/mm^2^ (ROC-AUC 0.93), while using *b* values ≤600 s/mm^2^, 91% sensitivity and 75% specificity were obtained (ROC-AUC 0.92). A summary of the recently published meta-analyses on breast DWI is reported in Table 1.

There is no clear consensus regarding the threshold values for ADC to diagnose malignancy. A substantial variability of threshold values, ranging 0.90–1.76 × 10^−3^ mm^2^/s has been observed (51). As a recommendation, a relatively higher threshold value may be used to minimize missing malignancy.

Of note, the studies evaluating mass lesions had a higher specificity (84%) than those evaluating non-mass lesions (70%) (2). When added to CE-MRI, DWI sequences showed a significant diagnostic gain: the summary ROC-AUC of CE-MRI combined with DWI was 0.94 compared with 0.85 for CE-MRI alone (2). Differently from 1H-MRS, contrast injection does not negatively impact on DWI performance (51); ADC measures were reported to not significantly change following DCE-MRI at 3 T, suggesting that DWI and DCE-MRI can be performed in any order without affecting diagnostic performance (52). Moreover, Janka et al. showed that DWI after contrast injection even leads to a slightly better benign/malignant discrimination (53).

Furthermore, DWI sequences are not dependent on CE-MRI. They can be performed without previous knowledge of lesion location, as the DWI field of view includes both breasts with a 4- to 5-min acquisition time.

Interestingly, some authors showed as DWI could be used as the main component of an unenhanced (*non-contrast*) MRI examinations with good sensitivity and high specificity, at least if we consider mass lesions. A reduced detection rate of small mass lesions and of non-mass lesions has to be taken into account (54, 55). However, a sensitivity theoretically comparable with that of screening mammography (54) as well as a potential for avoiding the double reading (55) candidate DWI as a sequence to be used for explorative non-contrast MRI screening studies.

Notably, DWI sequences are undergoing a progressive technical refinement. Spatial resolution and image quality are increasing, allowing also for a more detailed morphology evaluation (56, 57). As a matter of fact, authors have positively tried to integrate information coming from DWI into the Breast Imaging Reporting and Data System BI-RADS (58).

Considering the potential prognostic value of DWI, we finally highlight that a recent systematic review demonstrated that breast lesions with increased signal intensity on DWI and decreased signals on ADC are more likely associated to lymph-nodal metastases (59).

## Research Trends for 1H-MRS and DWI of the Breast

First of all, authors attempted to integrate CE-MRI, 1H-MRS, and DWI (multiparametric MRI, mpMRI) to yield significantly higher ROC-AUC (0.936) in comparison with just two of them (0.808) (60). Positron emission tomography (PET) was also added to provide an intriguing mpMRI/PET approach for lesion characterization (61), but we cannot propose this high-cost approach for characterization of breast lesions, when most cases can be solved by a needle sampling.

Regarding 1H-MRS, two interesting topics are multivoxel spectroscopy and statistical strategies for classifying MR spectra. Multivoxel 1H-MRS should overcome the need of *a priori* knowledge of lesion location (62), allowing for BC screening (or for stratifying BC risk) on the basis of tCho levels in the healthy gland tissue. However, multivoxel 1H-MRS is more technically challenging than single-voxel 1H-MRS for shimming and fat suppression (63). Statistical strategies for classification of spectra were proposed in a 2D correlated spectroscopy (COSY), where the composite resonances are separated by the use of a second frequency (63). However, this interesting approach seems to be not easy to be reproduced for clinical practice.

Finally, also 31P-MRS (64) was used to provide a direct method for the *in vivo* detection and quantification of endogenous biomarkers, yielding a new intriguing method for the non-invasive assessment of prognostic and predictive biomarkers in BC treatment.

Regarding DWI, several studies have shown that important information is lost when one relies just on the average ADC and that a higher sensitivity is given by minimum ADC instead of any metrics of the central tendency of ADC values distribution. Mori et al. (65) indicated that a minimum ADC value <1.1 × 10^−3^ mm^2^/s and that an ADC difference (maximum minus minimum ADC) greater than 0.23 × 10^−3^ mm^2^/s suggests the presence of invasive carcinoma in cases with only DCIS at biopsy, a crucial information for patient management.

Second, studies explored the possibility of characterizing axillary lymph nodes using DWI in BC patients. One study (66) considered only nodes with metastases ≥5 mm, obtaining 95% sensitivity and 92% specificity. More recent studies (67–69) obtained sensitivities from 72 to 85% and specificities from 80 to 88%. Future multidisciplinary researches should be placed in the context of the current rethinking of axillary dissection (70).

However, the most intriguing research areas for breast DWI are diffusion tensor imaging (DTI) and intravoxel incoherent motion (IVIM). For DTI (which adds information about tissue microstructure by addressing diffusion spatial direction), at least six DWI gradient directions should be applied, so that a symmetrical matrix, the diffusion tensor indeed, can be calculated, describing the anisotropic water diffusion in the tissue. Fractional anisotropy (FA) describes diffusion anisotropy quantitatively on a range from 1 (maximum anisotropy) to 0 (isotropy) (71). Experiences on breast DTI showed that FA does not have incremental value for differential diagnosis over ADC values (71). Eyal et al. (72) developed a breast DTI protocol at 3 T and image processing means for obtaining vector and parametric maps of the water diffusion tensor. Evaluation of the various diffusion parametric maps indicated that the prime diffusion coefficient and the maximal anisotropy are the most efficient parameters for detecting and diagnosing BC. So far, DTI turned out to be a great tool for visualizing the breast parenchyma but its clinical application remains to be investigated.

The IVIM quantifies molecular diffusion more accurately than ADC and provides additional information on microperfusion tissue properties. It separates the contribution of T2 from diffusivity using multiple *b* values, hence being less dependent on the choice of individual *b* values (73). However, IVIM as well as DTI imply longer acquisition times, and these techniques are not currently suitable for a large use in clinical practice.

## Conclusion

The results coming from the secondary evidence about 1H-MRS and DWI of the breast are clearly in favor of an easier and more effective use of DWI. If one of the two approaches for non-contrast breast MR has to be chosen for clinical practice, DWI is certainly the winner.

When looking at a clinical perspective, while 1H-MRS remained a tool with relevant limitations such as relatively long acquisition times, frequent low quality spectra, difficult standardization and quantification of tissue tCho concentration, DWI is feasible in almost all cases and adds diagnostic power to CE-MRI. Moreover, seminal studies showed the potential value of DWI as a stand-alone approach for BC detection.

As matter of fact, in the context of an ongoing international multicenter study exploring the value of preoperative CE-MRI with over 4500 patients enrolled so far (15), 84% of them were studied using a technical protocol including DWI among the standard sequences.

## Ethics Statement

Did the study presented in the manuscript involve human or animal subjects: Yes. The case presented in Figure 1 comes from a prospective clinical study approved by the Ethics Committee of the Azienda Ospedaliera Universitaria Integrata, Verona, Italy. The patient gave written informed consent.

## Author Contributions

FS is responsible for the conception of the work. LC and RT searched for literature focusing on secondary evidence. FS, LC, and RT drafted multiple versions of the manuscript. SM and CC revised the manuscript adding content of intellectual importance. All authors approved the final version of the manuscript.

## Conflict of Interest Statement

The authors declare that the research was conducted in the absence of any commercial or financial relationships that could be construed as a potential conflict of interest.
